# Supplementary material for: Generative diffusion surrogates with analytical variance schedule
Source: Nat Commun. 2026 Oct 8;17:10408. doi: 10.1038/s41467-026-77769-6 (PMC13649670; doi:10.1038/s41467-026-77769-6)
Supplement: Supplementary file 1 [file 41467_2026_77769_MOESM1_ESM.pdf]

nature portfolio

Peer Review File

Generative Diffusion Surrogates with Analytical Variance Schedule

Corresponding Author: Dr Patrick Reichherzer

This file contains all reviewer reports in order by version, followed by all author rebuttals in order by version.

Version 0:

Reviewer comments:

Reviewer #1

(Remarks to the Author)
Thank you for inviting me to evaluate the article titled "Generative Diffusion Surrogates with Analytical Variance Schedule". This work establishes a connection between macroscopic transport equations and the training schedules of diffusion models. By doing so, it eliminates the need for manual tuning, enabling alignment between the generative and physical timescales. The analytical enforcement of the variance path allows the network to focus on non-Gaussian structures, thereby facilitating accurate propagation of intermittency based on entrance statistics. The manuscript presents a novel approach by embedding the variance path into the diffusion model. However, there are several critical issues related to experimental validation, theoretical exposition, and clarity of writing that need to be addressed for the paper to meet the standards of publication. Specifically:

1. Abstract: The abstract lacks a clear description of the method's applicability and generalization. It also omits highlights and practical examples of its application.
2. Training and Sampling Costs: The manuscript does not provide a clear definition of the training and sampling costs (e.g., parameters, computational load) associated with introducing the variance path for finite-speed transport problems in the diffusion model. Additionally, the process of training and sampling for the score-based diffusion model needs to be detailed.
3. Lines 92-93: The reference format requires adjustment.
4. Lines 130-144: Equations (2) and (3) are repeated in Section 3.1. It is recommended that either this section or Section 3.1 provides a more concise summary of the forward and backward processes of the diffusion model.
5. Section 1.2: The concepts of "finite-speed transport" and "finite correlation time" should be explained more clearly, including why these factors impact the system's behavior.
6. Lines 208-211: A diagram should be included to illustrate the detailed training and inference phases.
7. Lines 227-229: The reason for introducing the condition "the analytic convolution is impractical" needs clarification.
8. Anchoring Physical Laws in the VE Strategy: Further details are needed on how physical laws are anchored within the VE strategy. Specifically, the manuscript should include relevant training and sampling details, such as an algorithm flowchart in the appendix. Additionally, comparative experiments (e.g., with and without physical laws) should be conducted to demonstrate that the generative model respects the physical timescales without the need for heuristic tuning.
9. Line 296: A more thorough explanation of the Central Limit Theorem is needed, particularly how it transforms the data into a Gaussian distribution.
10. Lines 297-298: Specific experimental verification is required to demonstrate that the physics-anchored VE model is optimal.
11. It is recommended to include additional experimental results or explore other application scenarios to showcase the method's applicability to different types of intermittent behaviors or physical systems.
12. The VE model is derived from Gaussian data, and while its effectiveness for non-Gaussian data has been experimentally verified, the manuscript lacks theoretical justification for its suitability and effectiveness in such cases.
13. Lines 348-350: Theoretical proof is needed to demonstrate that the network has successfully learned the score required to propagate the non-Gaussian structure inherited from the turbulent field.
14. Line 388: Can you provide examples that are applicable to other VE samplers?
15. Lines 407-411: A comparison with the case that does not incorporate physics-anchored input is necessary. This should be done by controlling variables (e.g., testing without physics-anchored input) to quantitatively evaluate the performance improvements provided by the physics-anchored input.
16. Lines 422-438: Only the summary comparison is provided here. The work should elaborate on the differences in flow

matching and other relevant aspects, along with experimental comparisons.
17. Uncertainty Analysis: As the diffusion model is probabilistic, it is crucial to assess the model's uncertainty (e.g., through multiple samplings). Please include uncertainty analysis results, using quantitative indicators such as variance, and provide related discussions in the turbulent plasma transport or modeling intermittency experiments.
18. Parameter Symbols: A table explaining the parameter symbols used in the Methods section would enhance accessibility for the readers.
19. A detailed derivation of Equation 11 is needed for clarity.
20. Please provide an explanation of how Equation 12 is derived from Equation 11.
21. The manuscript should define any symbols that appear for the first time. This should be verified throughout the entire paper.
22. The details of the diffusion model implementation, including training and sampling procedures, should be added to Section 3.4 for greater transparency.
23. More experimental comparisons with other diffusion models (e.g., DDPM, DDIM, EDM) should be provided to enhance the manuscript's experiments.
24. The derivations of the formulas are concise but could benefit from further explanations of key steps. For example, when deriving the diffusion coefficient (Eq. 17), the underlying physical mechanism should be explained, along with the rationale for the formula's validity.
25. The manuscript should clarify the details in Equations 18 and 19, particularly addressing why acceleration disappears in these equations.
26. Physical Background: Additional explanations of the physical background are necessary. For example, the terms "ballistic" and "diffusive" regions should be further elaborated to explain their physical significance. Furthermore, when discussing the spatial diffusion coefficient, the manuscript should clarify how it is extracted from experimental data or confirmed through experimental measurements. The concept of the high-stiffness limit and turbulent elements also requires more context—under what conditions does a particle enter the high-stiffness limit, and how does this limit affect transport?
27. Figures: The figures in the manuscript could be enhanced for greater clarity and visualization. For example, Figure 1 could provide more details about the training and inference process, while Figure 2 should briefly explain the specific meanings of the different lines (e.g., Fig. 2 b and c).
28. Figure 4 should include the uncertainty distribution of generated samples at different times.

Overall, while the methodology presented in the paper is interesting, particularly in terms of integrating the variance path into the diffusion model and addressing non-Gaussian structures, there are significant issues with the experimental validation, theoretical rigor, and model details that must be addressed.

(Remarks on code availability)

Reviewer #2

(Remarks to the Author)
SEE ATTACHMENT

(Remarks on code availability)
unfortunately, this is only a minimal code

Version 1:

Reviewer comments:

Reviewer #2

(Remarks to the Author)
The authors have improved the manuscript considerably and have addressed my concerns, so that I can suggest the manuscript for publication now.
I just have some minor remarks:

1. In Figure 2b–d, the authors show a black dotted line, which is said to represent the “analytical prediction of kurtosis” for the Kac process, analogous to the black dashed line given in Eq. (4).

It would be useful to know how the authors analytically compute the excess kurtosis for the Kac process (black dotted line), at least for the sake of reproducibility.

2. If everything is implemented correctly, the Goldstein paper [21] (I had no access) should contain the Kac kernel that leads to the Kac distribution described in our telegrapher’s equation paper. Nevertheless, including the kernel in an appendix would improve reproducibility.

3. Regarding the speed/damping parameters (a, c), I suspect that they were chosen to fit the variance curve in Fig. 2a. This would uniquely determine (a, c), and the excess kurtosis could indeed be negative. However, a clear explanation of how (a,

c) are selected would be desirable.

4. Finally, the missing purple curve (AI model variance) in Figure 3e is confusing. The authors state only that it reproduces the blue curve: “It reproduces the measured variance (e) and kurtosis evolution (f) without tuning.” However, the purple curve is shown only in Fig. 3f.

(Remarks on code availability)

Open Access This Peer Review File is licensed under a Creative Commons Attribution 4.0 International License, which permits use, sharing, adaptation, distribution and reproduction in any medium or format, as long as you give appropriate credit to the original author(s) and the source, provide a link to the Creative Commons license, and indicate if changes were made.
In cases where reviewers are anonymous, credit should be given to 'Anonymous Referee' and the source.
The images or other third party material in this Peer Review File are included in the article’s Creative Commons license, unless indicated otherwise in a credit line to the material. If material is not included in the article’s Creative Commons license and your intended use is not permitted by statutory regulation or exceeds the permitted use, you will need to obtain permission directly from the copyright holder.
To view a copy of this license, visit https://creativecommons.org/licenses/by/4.0/

Reviewer 1

Thank you for inviting me to evaluate the article titled "Generative Diffusion Surrogates with Analytical Variance Schedule". This work establishes a connection between macroscopic transport equations and the training schedules of diffusion models. By doing so, it eliminates the need for manual tuning, enabling alignment between the generative and physical timescales. The analytical enforcement of the variance path allows the network to focus on non-Gaussian structures, thereby facilitating accurate propagation of intermittency based on entrance statistics. The manuscript presents a novel approach by embedding the variance path into the diffusion model.

We would like to thank the reviewer for their valuable comments. These comments led to numerous improvements in the text, as well as the addition of a controlled comparison of different schedules (table 1), a formal optimality argument (methods, "Optimality within Gaussian surrogates"), and a complete description of the algorithm (Algorithm 1).

However, there are several critical issues related to experimental validation, theoretical exposition, and clarity of writing that need to be addressed for the paper to meet the standards of publication. Specifically:

1. Abstract: *The abstract lacks a clear description of the method's applicability and generalization. It also omits highlights and practical examples of its application.*

We have improved the abstract and highlighted that the important characteristics are that there is a macroscopic process that builds up as the sum of many independent microscopic processes. These systems can typically be described by the central limit theorem. In addition, the temporal evolution of the variance must be known. We have revised and quantified the schedule comparison and mention the result in the abstract.

2. Training and Sampling Costs: *The manuscript does not provide a clear definition of the training and sampling costs (e.g., parameters, computational load) associated with introducing the variance path for finite-speed transport problems in the diffusion model. Additionally, the process of training and sampling for the score-based diffusion model needs to be detailed.*

We have recorded the details of training and sampling in Algorithm 1. We have included a configuration table with important figures on the network architecture and the entire process. The computing costs are identical to the standard VE models. Except for the scalar schedule, nothing changes.

3. Lines 92-93: *The reference format requires adjustment.*

We have corrected the incorrect citations.

4. Lines 130-144: *Equations (2) and (3) are repeated in Section 3.1. It is recommended that either this section or Section 3.1 provides a more concise summary of the forward and backward processes of the diffusion model.*

In the methods section, we now only refer to the equations defined in the main text.

5. Section 1.2: *The concepts of "finite-speed transport" and "finite correlation time" should be explained more clearly, including why these factors impact the system's behavior.*

We have included an introduction to the results for this purpose. Here, the previously missing connection between the correlation length and finite-speed transport is now explained in more detail. The idea, especially with a view to later application, is that at scales below the correlation length, processes are often correlated and complex dynamics play a role. When these processes accumulate uncorrelated, the dynamics change to diffusive behavior, which flattens the variance curve.

6. Lines 208-211: *A diagram should be included to illustrate the detailed training and inference phases.*

We have expanded the caption of Figure 1 and now refer to Algorithm 1, which formally explains the steps.

7. Lines 227-229: *The reason for introducing the condition "the analytic convolution is impractical" needs clarification.*

The convolution formula in Equation 3 requires isotropic variance and a linear observation operator. This is not the case, for example, if there is location-dependent variance. However, the PF-ODE still provides a differentiable inference about the learned score.

8. Anchoring Physical Laws in the VE Strategy: *Further details are needed on how physical laws are anchored within the VE strategy. Specifically, the manuscript should include relevant training and sampling details, such as an algorithm flowchart in the appendix. Additionally, comparative experiments (e.g., with and without physical laws) should be conducted to demonstrate that the generative model respects the physical timescales without the need for heuristic tuning.*

Algorithm 1 now describes the detailed procedure. We have added a table for the hyperparameters. We have also expanded the comparative experiments (Figure 2 and linked table).

9. Line 296: *A more thorough explanation of the Central Limit Theorem is needed, particularly how it transforms the data into a Gaussian distribution.*

We have expanded the explanation in two places. In the new section at the beginning of the results, we explain that the correlation time represents a natural coarse-graining scale. For times well above the correlation time, many independent contributions add up, resulting in a Gaussian distribution according to the central limit theorem. In the plasma section, we explicitly distinguish between spatial averaging (the beam averages over many independent turbulent cells) and temporal accumulation (many successive scatterings) . Both lead to Gaussianization, but for different reasons.

10. Lines 297-298: *Specific experimental verification is required to demonstrate that the physics-anchored VE model is optimal.*

We have supplemented the empirical evidence by adding further schedulers and quantifying them in Table 1. The heuristic schedulers and the Kac model deviate significantly from the behavior defined by the CLT. We have also added error bands to the plots to account for uncertainties in the verification.

11. *It is recommended to include additional experimental results or explore other application scenarios to showcase the method's applicability to different types of intermittent behaviors or physical systems.*

In Figure 2, we now provide a generalized experiment with different target kurtosis. In addition to the plasma experiment, we show the Swiss roll as a non-physical experiment. Implementing one of the potential candidates now listed, such as anomalous intracellular transport, financial models with rough volatility, or transport in porous media, would go beyond the scope of this work.

12. *The VE model is derived from Gaussian data, and while its effectiveness for non-Gaussian data has been experimentally verified, the manuscript lacks theoretical justification for its suitability and effectiveness in such cases.*

We did not explain this point well in the previous draft and have now remedied this. We have added a new section in 1.1, which now explains that the non-Gaussian form is based on the microprocess and serves as the initial distribution. The duration of the microprocess is our coarse-graining time step. The VE now operates with this coarse-grained time scale and, based on the central limit theorem, relaxes the kurtosis toward a Gaussian distribution, but only over time, so that for early times, the distribution is not yet Gaussian, as shown more clearly in Figure 2.

13. Lines 348-350: *Theoretical proof is needed to demonstrate that the network has successfully learned the score required to propagate the non-Gaussian structure inherited from the turbulent field.*

A formal finite-sample proof is beyond current theory and we provide empirical validation instead.The kurtosis evolution (Equation 4) analytically predicts the relaxation. Figures 2b-d show that the generated samples reproduce this curve with less than 5 percent relative error (we determined this value in the linked code), for all readout times and for input kurtoses of 0.5, 1.0, and 1.5. The “Schedule ablation” table reports a reduced chi-square of about 1.48 for kurtosis across different input statistics. In the idealized limit of infinite data, denoising score matching should converge to the true score.

14. Line 388: *Can you provide examples that are applicable to other VE samplers?*

The schedule is a scalar path that can be used in any VE solver. The “Schedule ablation” table contains both PF-ODE lines (deterministic) and reverse SDE lines (stochastic) with the same schedule. Both deliver comparable results, confirming the independence from the

sampler. The discussion lists compatible solvers: Euler-Maruyama, Predictor-Corrector, Heun (second order), and the EDM framework. (Table “Schedule ablation”; Discussion.)

15. Lines 407-411: *A comparison with the case that does not incorporate physics-anchored input is necessary. This should be done by controlling variables (e.g., testing without physics-anchored input) to quantitatively evaluate the performance improvements provided by the physics-anchored input.*

The table 1 now shows the comparison for a CLT-dominated process. Architecture, optimizer, data, and computing budget are identical. Only the schedule varies. The reduced chi-square quantifies the difference.

16. Lines 422-438: *Only the summary comparison is provided here. The work should elaborate on the differences in flow matching and other relevant aspects, along with experimental comparisons.*

We implemented the Kac flow baseline and presented it in Figure 2 and Table 1. As expected, the Kac flow also approximately recovers the telegraph variance path (chi-squared 2.09). However, the kurtosis deviates significantly (chi-squared 73.73) because this process differs in that it causes a sub-Gaussian distribution (negative kurtosis) due to the transition from ballistic to diffusive behavior. In contrast, our plasma data show consistently positive kurtosis (Figure 3f) because the non-Gaussianity comes from the initial statistics, the non-Gaussian microprocess. One possible scenario in which the Kac flow could be ideal is if, for example, particles with significantly lower energies and stronger scattering processes were used in the plasma laboratory experiment and it were possible to measure in the middle of the plasma on scales below the correlation length. Since this is not practical, but rather the beam is measured after it has passed through the plasma, i.e., on scales larger than the correlation length, the Kac process is only an interesting thought experiment for this application for the possible description of the microprocess for low energies on smaller scales. This is because low-energy particles would already become diffusive on smaller scales, i.e., already below the correlation scale relevant for high-energy particles.

17. Uncertainty Analysis: *As the diffusion model is probabilistic, it is crucial to assess the model's uncertainty (e.g., through multiple samplings). Please include uncertainty analysis results, using quantitative indicators such as variance, and provide related discussions in the turbulent plasma transport or modeling intermittency experiments.*

We now report the mean plus/minus one standard deviation from 10 independent models in Figure 2 (shaded bands) and from 5 sampling seeds in Figure 4 (shaded bands). For plasma intermittency, the empirical kurtosis gap is 0.02 plus/minus 0.08, which is consistent with zero within the statistical uncertainty.

18. Parameter Symbols: *A table explaining the parameter symbols used in the Methods section would enhance accessibility for the readers.*

We have now added Table 2 for this purpose. It lists symbols, meanings, units, and the location of first occurrence. It is referenced at the beginning of the methods section.

19. *A detailed derivation of Equation 11 is needed for clarity.*

Section 3.2 “Optimality within Gaussian surrogates” of the methods section now explicitly explains the W_2 coupling. If one takes an optimal coupling of the two kernels and adds a common random variable X_0 independently. Because X_0 is eliminated in the difference, only the distance between the kernels remains. (Note: Old Eqs. 11 & 12 are now Eqs. 9 & 10 in the revised manuscript)

20. *Please provide an explanation of how Equation 12 is derived from Equation 11.*

We have now added the transition between the equations. The first kernel is set equal to the physical transport kernel and the second kernel is set equal to the Gaussian surrogate kernel. Inserting this into the contraction inequality directly yields the bound.

21. *The manuscript should define any symbols that appear for the first time. This should be verified throughout the entire paper.*

We have inserted definitions directly into the text, for example, the gyro radius $r_g = mv/(qB)$ in the plasma section and the kurtosis K = fourth cumulant divided by variance squared in the CLT section. In Table 2, we now also list the first mention of the quantity in the text.

22. *The details of the diffusion model implementation, including training and sampling procedures, should be added to Section 3.4 for greater transparency.*

Section 3.4 now describes the architecture (10-block FiLM-MLP with GroupNorm, approximately 1.16 million parameters), the optimizer (AdamW, learning rate 0.001), and the sampling (Euler PF-ODE, 500 steps). Algorithm 1 and the configuration table complete the description.

23. *More experimental comparisons with other diffusion models (e.g., DDPM, DDIM, EDM) should be provided to enhance the manuscript's experiments.*

We have included a Karras/EDM baseline in the “Schedule ablation” table. VP-based models such as DDPM and DDIM are structurally unsuitable because their mean-reverting drift limits the variance to one, which would require unphysical rescaling. In physical models, variance increases over time, as in VE. For example, the physical process of the beam passing through plasma has an unbounded growing variance, which fits the VE framework. Our comparisons are therefore limited to VE baselines to isolate the effect of the schedule within the same SDE class.

24. *The derivations of the formulas are concise but could benefit from further explanations of key steps. For example, when deriving the diffusion coefficient (Eq. 17), the underlying physical mechanism should be explained, along with the rationale for the formula's validity.*

We have expanded the section on particle transport. It now includes the Taylor-Green-Kubo double integral and the derivation of both limiting cases. For short times (well below the

correlation time), the autocorrelation is nearly constant and the variance grows quadratically (ballistically).
For long times (well above the correlation time), the integral saturates and the variance grows linearly (diffusively), controlled by the velocity space diffusion coefficient D_v.

25. *The manuscript should clarify the details in Equations 18 and 19, particularly addressing why acceleration disappears in these equations.*

We have added an explanatory sentence explaining that the mean square acceleration is pulled into the diffusion coefficient D_v via the Green-Kubo integral. This is standard notation. The spatial diffusion coefficient kappa still contains the acceleration dependence, which is not obvious but is now explicitly mentioned.

26. Physical Background: *Additional explanations of the physical background are necessary. For example, the terms "ballistic" and "diffusive" regions should be further elaborated to explain their physical significance. Furthermore, when discussing the spatial diffusion coefficient, the manuscript should clarify how it is extracted from experimental data or confirmed through experimental measurements. The concept of the high-stiffness limit and turbulent elements also requires more context—under what conditions does a particle enter the high-stiffness limit, and how does this limit affect transport?*

Section 1.2 explains the two regimes via persistent and decorrelated correlations, respectively. The plasma section defines the limiting case where the gyro radius is much larger than the correlation length. The methods section shows two equivalent ways to determine kappa: (i) from the long-term slope of the displacement variance, (ii) from the measured D_v via small-angle scattering.

27. Figures: *The figures in the manuscript could be enhanced for greater clarity and visualization. For example, Figure 1 could provide more details about the training and inference process, while Figure 2 should briefly explain the specific meanings of the different lines (e.g., Fig. 2 b and c).*

We have revised the captions for Figures 1 and 2 and completely reworked Figure 2 after making the further comparisons requested in the previous comments. Each sub-figure is now described individually. Figure 1 refers to Algorithm 1. Figure 2 explains each timeline, the content of each sub-figure, and the uncertainty bands. We have also added error bands to show the uncertainties in the figures.

28. *Figure 4 should include the uncertainty distribution of generated samples at different times.*

We have added uncertainty bands (plus/minus one standard deviation over 5 seeds) to the precision-recall curves in Figure 4. Figure 2 also shows uncertainty bands (10 seeds).

Overall, while the methodology presented in the paper is interesting, particularly in terms of integrating the variance path into the diffusion model and addressing non-Gaussian

structures, there are significant issues with the experimental validation, theoretical rigor, and model details that must be addressed.

Reviewer 2

The authors aim to simulate the behavior of certain physical systems (e.g. charged particles in a turbulent magnetic field) with a ballistic-to-diffusive transition, i.e. ... as given by the telegraph regime with time set by .... The macroscopic behavior bridging these regimes is given by the telegraph equation which variance evolution is known as ..., where C is such that .... The authors propose to learn a diffusion model with a schedule adapted to ..., more precisely ... (forward SDE - zero drift), ... (reverse SDE) where ....
I am not a physicist, so that I cannot evaluate the physical relevance of the work. But to the best of my knowledge the approach is new and I found the paper overall interesting so that it may merit publication in Nature Communications. Besides the small issues below which should be addressed, my main question is, why the authors do not simulate the telegrapher model by a non-Gaussian path as in reference [13]?

We thank the reviewer for the constructive evaluation. We have now included this non-Gaussian telegraph baseline (Kac flow) and show that it does not reproduce the observed sign of kurtosis during the transition. Specifically, we have added a Kac flow baseline that satisfies the same telegraph variance path (table "Schedule ablation"). The Kac kernel has a limited carrier (it cannot extend beyond the finite propagation speed times the time). As a result, it temporarily generates sub-Gaussian intermediate distributions with negative kurtosis during the transition from ballistic to diffusive. However, our plasma data show consistently positive kurtosis (Figure 3f). This is because the non-Gaussianity is inherited from the intermittent input statistics and is not generated by the transport kernel. The Gaussian VE surrogate naturally relaxes a positive input kurtosis to zero through convolution. It is therefore the physically correct choice in this regime, which is dominated by the central limit theorem. We agree that the Kac kernel is compelling in regimes where limited propagation speed dominates the physics. We have included this as a complementary approach in the discussion. ("Entrance-only training"; table "Schedule ablation"; discussion.)

In addition to the minimal notebook already provided, we have now added the complete code base, which contains the ablation scripts, plasma simulation pipelines, the Swiss Roll notebook, pre-calculated outputs, dependency specifications, and quick start instructions (see README). A public repository will be created upon publication.

1. *p. 2 lines 91-93: here something is wrong with the references*

Fixed in the second paragraph of the introduction.

2. *p. 2 lines 96-99 'Nevertheless ...' This is not true. The proposed method needs the same things than e.g. flow matching models, for an overview we refer to C Wald, G Steidl, Flow Matching: Markov kernels, stochastic processes and transport plans, in: Variational and Information Flows in Machine Learning and Optimal Transport, Springer 2025*

We agree that the previous wording was too vague. We have revised the text to describe the difference structurally. Our approach does not learn a time-dependent transport map. Instead, it imprints the physical clock by anchoring the variance path and trains a score model on the resulting anchored marginal distributions. Flow-based and trajectory-based approaches (including flow matching) learn or prescribe a time-parameterized velocity field along a selected path between endpoint distributions. We have added the suggested reference Wald and Steidl (2025).

***3.** Kurtosis could be earlier introduced than just in Sec. 3.3. or at least one could refer to Sec 3.3 when it appears first*

Kurtosis is now defined at its first appearance in the CLT section. This definition precedes the kurtosis evolution equation.

***4.** I had some problems with the figures; maybe because several figures have the same caption. Could this be relaxed?*

We fully revised Fig. 2 and all captions are now independent with unique bold titles. Each subfigure is described individually in each caption.

***5.** I guess in Fig. 4 bottom (text above the figure: it took me a while that these things are indeed in a row - please improve); the figure has no scales. Further the reason for this ablation study became not quite clear to me. The spiral of the telegraph approach appears to be quite blurry and the cosine seems to do a better job in generating samples from the spiral target distribution.*

We have replaced Figure 4 with higher-resolution partial illustrations and added axis scales. The visual differences in the telegraph in the early stages can be explained by the slower noise reduction in the ballistic phase. However, when convergence is achieved, the telegraph achieves a better precision-recall balance: at k equal to 5, the telegraph achieves a recall of 0.83 compared to 0.72 for the cosine with comparable precision (0.43 compared to 0.44). For k greater than 10, telegraph provides the best overall precision–recall balance, and cosine can have slightly higher precision at intermediate neighborhood sizes (e.g., k=20), while telegraph achieves higher recall at smaller k and becomes comparable/superior as k increases.These numerical values are now included in the text. We have also added uncertainty bands (plus/minus one standard deviation over 5 seeds). We have also better explained the purpose of this study, as we wanted to show an experiment that tests the schedule independently of physics.

***6**. I do not like it that several formulas are so often repeated instead of referring to some number, e.g. the equation in (2) appears at several places*

All duplicate equations in the methods section have been removed and replaced with cross-references to the equation numbers in the main text.

We thank the reviewer for the positive assessment and these helpful final comments. We have addressed all four points. In particular, we added a dedicated Methods subsection describing the full Kac kernel, its moments and parameter selection, and clarified the corresponding Results discussion and figure captions.

1. *In Figure 2b–d, the authors show a black dotted line, which is said to represent the "analytical prediction of kurtosis" for the Kac process, analogous to the black dashed line given in Eq. (4). It would be useful to know how the authors analytically compute the excess kurtosis for the Kac process (black dotted line), at least for the sake of reproducibility.*

Thank you, we now explain how we compute the Kac excess kurtosis from the second and fourth moments of the full kernel, including its two endpoint point masses. Methods §4.4 now describes the kernel variance and excess kurtosis in Eqs. (13) and (14), and the resulting marginal excess kurtosis after the convolution with the entrance distribution in Eq. (15). Equation (15) gives the dotted reference in Fig. 2b–d and is cited directly in the caption.

2. *If everything is implemented correctly, the Goldstein paper [21] (I had no access) should contain the Kac kernel that leads to the Kac distribution described in our telegrapher's equation paper. Nevertheless, including the kernel in an appendix would improve reproducibility.*

We have included the full Goldstein–Kac density in Methods §4.4, Eq. (12). This includes the singular endpoint contributions, continuous Bessel-function contribution and bounded support. We placed it in Methods so that the kernel, moments and parameter choice can be found together.

3. *Regarding the speed/damping parameters (a, c), I suspect that they were chosen to fit the variance curve in Fig. 2a. This would uniquely determine (a, c), and the excess kurtosis could indeed be negative. However, a clear explanation of how (a, c) are selected would be desirable.*

We agree and added the missing required clarification. Indeed, the parameters are not fitted. They are fixed by requiring the Kac process to reproduce the anchored telegraph variance path. Then, the Kac variance in Eq. (13) reduces to the telegraph variance in Eq. (6). In the Results, we now note that identical variance evolution does not determine higher moments, propagators, or memory.

4. *Finally, the missing purple curve (AI model variance) in Figure 3e is confusing. The authors state only that it reproduces the blue curve: "It reproduces the measured variance (e) and kurtosis evolution (f) without tuning." However, the purple curve is shown only in Fig. 3f.*

We agree that the previous presentation was confusing and improved it accordingly. The former Fig. 3 is now Fig. 4. We now explain in both the text and caption that the model variance in panel e is the imposed anchored variance path and is therefore not plotted as a separate purple curve. We show the purple curve only in panel f, because it represents the non-trivial sampled prediction of a higher moment. The wording of the corresponding claim has been revised accordingly.
